# Lymphangiogenesis in gastric cancer: function and mechanism

**DOI:** 10.1186/s40001-023-01298-x

**Published:** 2023-10-07

**Authors:** Pengpeng Liu, Ping’an Ding, Chenyu Sun, Shuya Chen, Scott Lowe, Lingjiao Meng, Qun Zhao

**Affiliations:** 1 The Third Department of Surgery, The Fourth Hospital of Hebei Medical University, Shijiazhuang, 050011 Hebei China; 2 Hebei Key Laboratory of Precision Diagnosis and Comprehensive Treatment of Gastric Cancer, Shijiazhuang, 050011 China; 3 AMITA Health Saint Joseph Hospital Chicago, 2900 N. Lake Shore Drive, Chicago, IL 60657 USA; 4 Newham University Hospital, Glen Road, Plaistow, London, E13 8SL England, UK; 5 College of Osteopathic Medicine, Kansas City University, 1750 Independence Ave, Kansas City, MO 64106 USA; 6 Research Center of the Fourth Hospital of Hebei Medical University, Shijiazhuang, 050011 China

**Keywords:** Gastric cancer, Lymphangiogenesis, Lymph node metastasis, Therapeutics

## Abstract

Increased lymphangiogenesis and lymph node (LN) metastasis are thought to be important steps in cancer metastasis, and are associated with patient's poor prognosis. There is increasing evidence that the lymphatic system may play a crucial role in regulating tumor immune response and limiting tumor metastasis, since tumor lymphangiogenesis is more prominent in tumor metastasis and diffusion. Lymphangiogenesis takes place in embryonic development, wound healing, and a variety of pathological conditions, including tumors. Tumor cells and tumor microenvironment cells generate growth factors (such as lymphangiogenesis factor VEGF-C/D), which can promote lymphangiogenesis, thereby inducing the metastasis and diffusion of tumor cells. Nevertheless, the current research on lymphangiogenesis in gastric cancer is relatively scattered and lacks a comprehensive understanding. Therefore, in this review, we aim to provide a detailed perspective on molecules and signal transduction pathways that regulate gastric cancer lymphogenesis, which may provide new insights for the diagnosis and treatment of cancer.

## Introduction

Gastric cancer (GC) is the third leading cause of cancer-related death worldwide, and its incidence varies by gender and region. The prevalence rate is higher in East Asia, and men are more likely to get sick than women [1]. The latest statistics show that there are more than 1 million patients with GC worldwide, and about 770,000 patients died of GC. Although the incidence of GC has declined, it remains a major global health problem [2]. There are many risk factors for GC, such as *Helicobacter pylori* infection, drinking, smoking, high-salt diet, EBV infection and hereditary family history. Its occurrence is closely related to precancerous lesions such as intestinal metaplasia, chronic atrophic gastritis and atypical hyperplasia [3]. For early gastric cancer (EGC) patients with low TNM stage and no LN metastasis, endoscopic mucosal resection (EMR) can achieve clinical cure [4]. For newly diagnosed resectable advanced gastric cancer (AGC) patients, the standard treatment is gastrectomy plus D2 LN dissection combined with postoperative adjuvant chemotherapy. However, for patients with resectable or unresectable AGC who have a late initial stage (clinical stage III and above), preoperative neoadjuvant therapy (standard chemotherapy regimen combined with molecular targeted therapy or immunotherapy, etc.) is used to reduce tumor staging, improve surgical success, and prolong patient survival [5, 6]. The 5-year survival rate of EGC is more than 90% after systemic treatment [7]. Considering the strong concealment of EGC and lack of early screening for GC susceptible population, more than 70% of patients show advanced disease at the time of initial diagnosis, and about 90% of patients with advanced gastric cancer die from primary tumor metastasis [8].

The clinical prognosis and survival time of tumor patients mainly depend on the local or distant metastasis caused by the primary tumor, and the invasion of regional LNs or sentinel lymph nodes (SLN) is considered to be a key factor contributing to the patients’ poor prognosis [9]. Although it is well established that metastasis of tumor cells is mainly through lymphatic vessels and blood vessels, fewer studies have been done on lymphatic pathways when compared to vascular pathways. Thus, it is necessary to understand the mechanism of tumor lymphatic metastasis at the molecular level for better tumor treatment. Previous studies have shown that lymphatic vessels undergo dynamic changes during tumor metastasis, and the formation of new lymphatic vessels and the remodeling of existing lymphatic vessels are considered to be important steps in cancer metastasis [10]. Moreover, recent studies have also found that tumor LN colonization can induce tumor immune tolerance and promote distant metastasis [11]. Hence, recognizing the potential functions of LN invasion and lymphangiogenesis in cancer can achieve an effective therapeutic strategy to limit tumor metastasis and diffusion by targeting blocking lymphangiogenesis signaling pathways and key inducing molecules. In this review, we aim to present some constructive knowledge on the essential molecules and signaling pathways that regulate lymphangiogenesis in GC. These findings might provide insights into new directions for cancer research, diagnosis, and potential treatment options and in future.

### Structure and function of lymphatic system

**Fig. 1:**
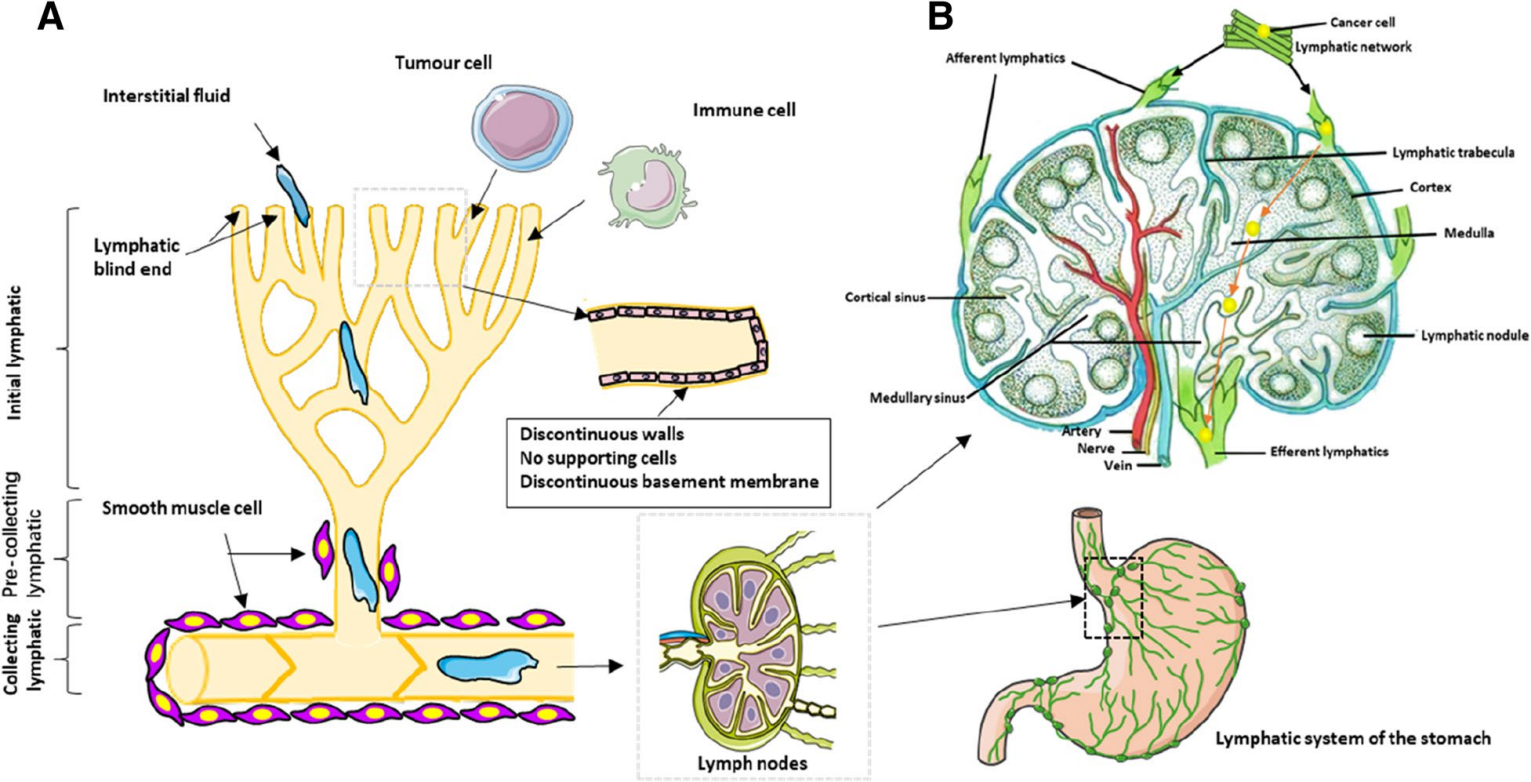
Represents the structure of the lymphatic system and tumor cells entering and leaving the draining LNs. **a** represents the hierarchical structure of lymphatic subtypes. Various cells (including cancer cells and immune cells) derived from the tumor microenvironment enter the LNs with the interstitial fluid passing through the initial lymphatic, the pre-collective lymphatic and the collecting lymphatic in turn. **b** represents the cancer cells into and out of the drainage LNs. With the interstitial fluid, the cancer cells begin at the blind end of the lymphatic, and then enter the afferent lymphatic, medullary sinus and efferent lymphatic of the draining LNs in turn, and finally the efferent lymphatic become the afferent lymphatic of other LNs

The lymphatic system is essential for regulating immune function, stabilizing tissue fluids, and inflammatory responses [10]. Lymph fluids carrying cells and antigens enter and leave the draining LNs mainly through the subcapsular, cortical and medullary sinus systems. In physiological conditions such as inflammation and cancer, the lymphatic sinus system plays a pivotal role in regulating immune functions, which it does so by changing the state of lymphatic endothelial cells (LECs). As a selective semi-permeable barrier, LECs serve not only as a sorting agent for cells and antigens in LN parenchyma, but also act as antigen-presenting cells. LECs are primarily generated by venous endothelial cells through vascular germination and form rich lymphatic networks in tissues. The network begins at the blind end of the lymphatic vessels, and then converges on the afferent lymphatic vessels of the draining LNs. Subsequently, the lymphatic network forms a medullary sinus at the LN portal, and finally the dense medullary sinus network converges into a single efferent lymphatic vessel [12] (Fig. 1B).

The initial lymphatic vessels are usually manifested as blind tubes with fewer branches and valveless structures [10, 13]. Electron microscopy showed that the initial lymphatic vessels usually had the following characteristics: irregular lumen, discontinuous basal layer and no pericytes, but with LEC, anchor wires and initial junction complex, etc. The anchor wire can connect LEC with elastic fibers in the tissue. The connection between LEC and elastic fibers and the unique discontinuous cell–cell junction between LECs allow tissue fluid to enter the lymphatic caecum through the vascular valve. Subsequently, the lymph flows through the deep anterior collecting duct into the collecting lymphatic vessels (characterized by the presence of basement membrane, flow-regulating valves, and surrounding VSMC layers), and finally returns to the blood vessels through the thoracic duct [10, 14]. However, when the lymph flows through the collecting lymphatic vessels, it flows through the LNs (Fig. 1A).

The gastric lymphatic network usually starts from the surface, internal and inferior vascular plexus of the muscularis mucosa and is widely distributed in all layers of the gastric wall [15]. Many capillary lymphatic with blind ends are evenly distributed in the gastric mucosa, which are usually located at the base of the gastric gland and have no obvious valvular structure. However, the lymphatic vessels in the gastric submucosa usually have a typical blind end and valvular structure. Mucosal lymphatic vessels establish a common outflow tract between the mucosa and the submucosa, allowing small lymphatic vessels in the mucosa to flow directly or through trafficking branches into the submucosa. In addition, the distribution of muscular lymphatic vessels is extremely irregular and intertwines in muscle bundles, while lots of lymphatic vessels in the submucosa can enter the serosa through the muscle bundles. Therefore, the abundant lymphatic network in the serosa forms an effective extraorgan lymphatic drainage pathway.

In patients with severe atrophic gastritis, gastric mucosal surface epithelial height is significantly reduced and the abnormal lymphatic vessels can be found, which may lead to atypical cells easily entering the lymphatic circulation and LN metastasis in EGC. In addition, another possible cause of LN metastasis in EGC is tumor cell proliferation induced by lymph circulation disorder. Since the initial lymphatic vessels lack a complete basal layer, the dilated lymphatic vessels caused by lymphatic circulation disorders are easily invaded by tumor cells [16]. Moreover, different types of lymphatic vessels may be affected by tumor-derived growth factors in cancer patients, leading to the regulation of lymphangiogenesis and immune function, and all of which may increase the metastasis of tumor cells to LN and may metastasize to distant organs [11]. Consequently, the establishment of sensitive lymphangiogenesis markers is extremely important for accurately identifying the early stages of tumor lymph node invasion and tumor-derived lymphangiogenesis.

### Lymphatic markers of tumor-associated lymphangiogenesis

Like angiogenesis, lymphangiogenesis also requires a series of cellular processes, including proliferation, germination, migration and tube formation [10, 13]. The key to lymphangiogenesis is the proliferation and migration of LECs, and LECs play an active role in the interaction between tumor cells and lymphatic vessels and in the formation of LN organs [10, 12, 13]. Besides, lymphatic markers of LECs have been used to identify lymphatic dysfunction and tumor-associated lymphangiogenesis. For example, lymphatic hyaluronic acid receptor 1 (LYVE1), Prospero homeobox 1 protein (Prox1), SOX18, neuropilin protein 2 (NRP-2), podoplanin (PDPN) and vascular endothelial growth factor 3 (VEGFR3) [10, 13]. However, LYVE1 and PDPN are the two most commonly used lymphatic markers [10], and its antibodies can be used to identify lymphatic vessels in human or animal experimental tumors by immunohistochemistry or immunofluorescence. Studies have shown that tumor lymphatic vessels may increase (LEC proliferation) under the action of lymphangiogenesis factors (such as VEGFC or VEGFD), and the large contact area between lymphatic vessels and tumor cells is believed to contribute to tumor cells entering the lymphatic vessels thereby promotes tumor metastasis and diffusion. On the contrary, studies in animal models have shown that although lymphangiogenesis provides a prerequisite for lymphatic invasion and metastasis, it might not be necessary for LN metastasis of tumor cells. Therefore, though it is undeniable that many studies have shown that lymphangiogenesis is considered to play an indispensable role in tumor LN metastasis, this process seems to have a complex underlying mechanism [17, 18].

### Signal transduction pathway related to lymphangiogenesis

**Fig. 2:**
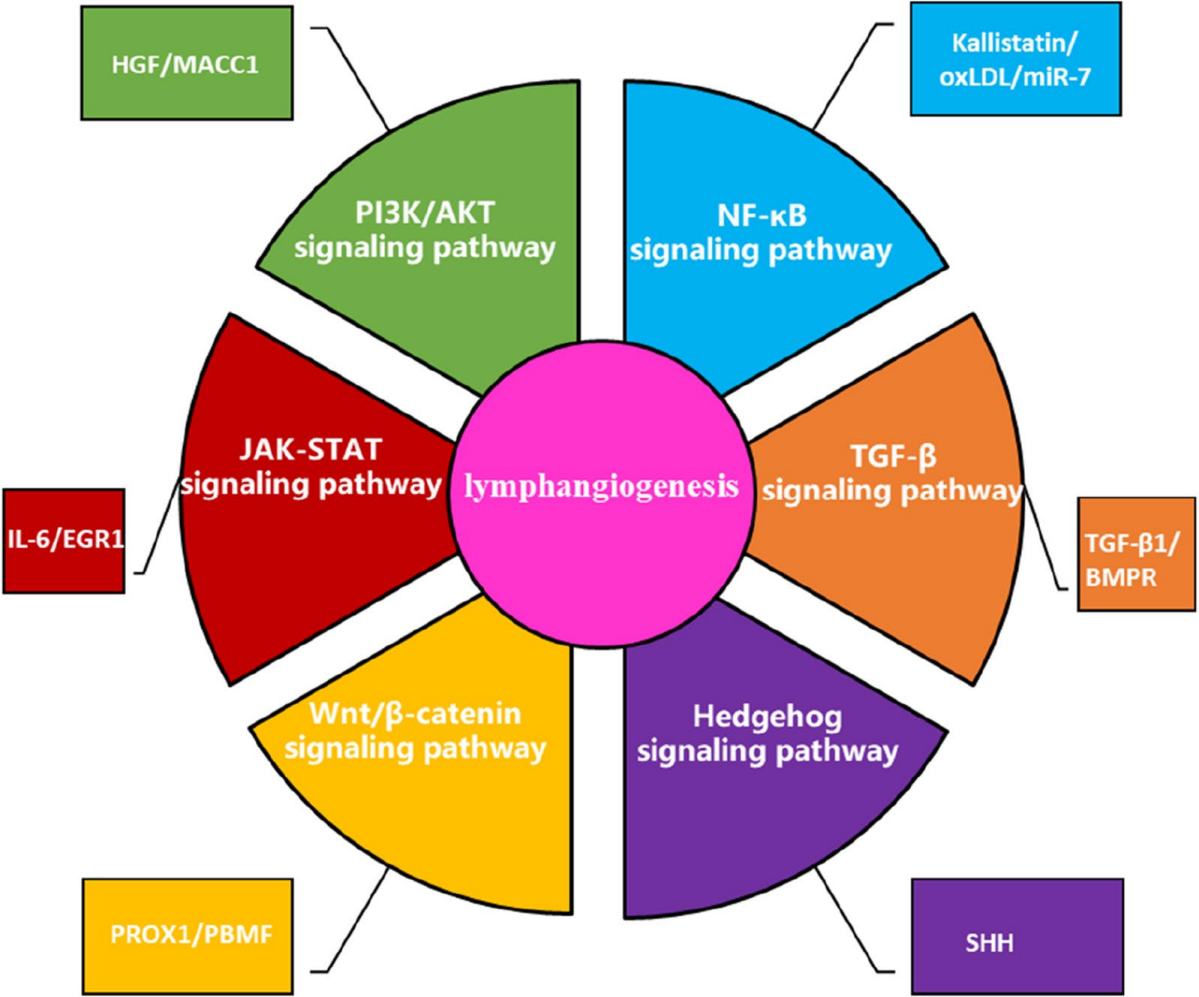
Represents the signaling pathways that may be involved in lymphangiogenesis

The VEGFC/D-VEGFR3 axis is primarily activated by proteolysis to promote tumor-associated lymphangiogenesis and metastasis to the lymph nodes. VEGFC and VEGFD are usually expressed in primary human tumors or their related matrix and are secreted by tumor cells, immune cells and tumor-associated fibroblasts, while VEGFR3 is mainly expressed in LECs [10]. Studies have shown that anti-VEGFR3-specific monoclonal antibodies (mAbs) can limit tumor lymphangiogenesis and LN metastasis [10, 19]. Neurogenin (NRP2) is a transmembrane signaling protein and a co-receptor of the VEGF family, which is coupled with VEGFR3 and mediates VEGF-C-induced lymphatic sprouting [20]. Blocking NRP2 can prevent LEC migration, reduce lymphangiogenesis and decrease the incidence of LN metastases [21] (Fig. 2).

However, tumor lymphangiogenesis is usually the result of multiple factors. So it is a very popular research topic to further explore the upstream signaling mechanism for finding effective therapeutic targets for LN metastasis and lymphangiogenesis. Here, we introduce and discuss the following pathways, and lymphangiogenesis-related factors based on recent studies on lymphangiogenesis:

1. PI3K/AKT signaling pathway [22]: Phosphatidylinositol 3-kinase (PI3K)/protein kinase B (AKT) signaling pathway is one of the most important signaling pathways in cells. Its main role is to inhibit apoptosis and promote proliferation. The PI3K/AKT-mediated mTOR signaling pathway is aberrantly regulated in a variety of malignant tumors, promotes tumor cell proliferation and neovascularization, and is closely related to tumor invasion and metastasis. Mechanism studies have shown that Akt/mTOR signaling axis can mediate VEGF-C/D secretion to participate in and regulate lymphangiogenesis in GC. The protein expression of p-Akt and p-mTOR were positively correlated with the expression of VEGF-C and VEGF-D in GC tissues and cells, and inhibition of p-Akt and p-mTOR significantly reduced VEGF-C and VEGF-D expression [23]. Yan et al. found that miR-182-5p directly targets VEGF-C and regulates lymphangiogenesis in colon cancer through ERK and AKT signaling pathways [24]. Hepatocyte growth factor (HGF) has been shown to stimulate the proliferation, tube formation and migration of LECs through downstream ERK1 and PI3K signals, while the HGF/c-Met signal transduction axis is associated with tumor lymphangiogenesis [25, 26]. In addition, MACC1 can activate HGF/c-Met signaling pathway and upregulate the expression of VEGF-C/D, thereby promoting lymphangiogenesis and LN metastasis [27].
2. Hedgehog signaling pathway [28]: Hedgehog (Hh) signaling molecule is a localized protein ligand secreted by signal cells. Hedgehog controls cell growth, proliferation and differentiation during development. When the Hedgehog pathway is abnormally activated, it may induce the occurrence and development of tumors. Hedgehog has three homologous genes: Sonic Hedgehog (SHH), Indian Hedgehog (IHH) and Desert Hedgehog (DHH), which encode SHH, IHH and DHH proteins, respectively. Lee et al. [29] showed that the expression of Shh was positively correlated with LN metastasis, high lymphatic vessel density and poor prognosis by immunohistochemical analysis of 178 cases of GC. Mechanistically, SHH can induce epithelial–mesenchymal transition (EMT), matrix metalloproteinase 9 (MMP-9) activity and tumor lymphangiogenesis through the PI3K/Akt pathway, thereby promoting tumor progression and LN metastasis. Besides, SHH can also regulate lymphangiogenesis in pancreatic cancer [30]. Hedgehog signal was enriched in breast cancer intratumoral lymphatic endothelial cells (iLECs) based on cancer stem cell-related gene sets [31].
3. NF-κB signaling pathway [32]: NF-κB (nuclear factor-activated B cell κ-light chain enhancement) is a protein complex that is widely used as a gene regulator to control cell proliferation and cell survival. NF-κB can be involved in the regulation of VEGF expression, and sustained activation of NF-κB can enhance VEGF gene transcription [33]. Cellular inhibitor of apoptosis 2 (cIAP2) is one of the most widely studied human IAPs. The expression of cIAP2 is increased in gallbladder cancer (GBC) and is related to the prognosis of patients. In addition, c IAP2 was identified as a lymphangiogenesis factor in GBC cells, thereby promoting LN metastasis of GBC cells [34]. In addition, Integrins, RIP1 and HN1 also promote tumor-associated lymphangiogenesis and LN metastasis by activating the NF-kappaB signaling pathway [35–37].
4. TGF-β signaling pathway [38]: The transforming growth factor-β (TGF-β) pathway is involved in many cellular processes in both mature organisms and developing embryos, including cell growth, cell differentiation, apoptosis, cell homeostasis and other cellular functions. However, multicenter transcriptome and cancer genome mapping studies have shown that TGF-β may also play an important role in LN metastasis and lymphangiogenesis [10, 39, 40]. For example, TGF-β1 can activate Smad pathway to regulate the expression of VEGF-C and participate in tumor lymphangiogenesis. In addition, tube formation assay and tumor xenograft mouse model also confirmed that TGF-β1 increased lymphangiogenesis, while inhibition of TGF-α1 blocked lymphangiogenesis [41]. Bone morphogenetic protein (BMP) is a member of TGF-β, which is also involved in the occurrence and progression of malignant tumors. Analyzed of the expression of BMP and its receptor (BMPR) based on TCGA GC database and GEO database found that high BMPR expression was highly correlated with tumor-related lymphangiogenesis and was involved in promoting tumor growth, expansion and diffusion [42].
5. JAK–STAT signal pathway [43]: The Janus kinase/signal transducer and activator of transcription (JAK/STAT) signaling pathway is a common pathway for many cytokine signal transduction, which is widely involved in cell proliferation, differentiation, apoptosis and inflammation processes. For example, IL-6-mediated JAK–STAT3/VEGF-C signaling pathway can promote tumor growth, invasion and lymphangiogenesis [44]. Furthermore, a study based on human skin lymphatic endothelial cells (HDLEC) showed that ERG1 can promote lymphangiogenesis by activating the SOX18/JAK/STAT3 cascade [45]. As a transcription factor that binds to the promoter, EGR1 is considered to be a therapeutic target for many diseases [46]. And SOX18, a downstream factor of EGR1, can promote tumor-induced lymphangiogenesis [47, 48].
6. Wnt/β-catenin signaling pathway [49]: Wnt is a secreted glycoprotein that interacts with specific receptors on the cell surface and cause β-Catenin accumulation through a series of phosphorylation and dephosphorylation processes of downstream proteins. As a multifunctional protein, β-Catenin interacts with E-Cadherin at cell junctions and participates in the formation of adhesive bands. Free β-Catenin enters the nucleus to regulate gene expression, and its abnormal expression or activation can induce tumorigenesis. However, typical Wnt/β-catenin signaling is also necessary for lymphangiogenesis. For example, research by Cha et al. showed that the oscillatory shear stress (OSS) that promotes lymphatic maturation can activate Wnt/β-catenin signaling, which in turn activates FOXC2 to regulate lymphatic development [50]. Wnt/β-catenin signaling is also involved in the regulation of VEGF-C/D-VEGFR-3 expression. Such as PBMF can induce tumor epithelial–mesenchymal transition (EMT) and lymphangiogenesis by regulating Wnt/β-catenin signaling pathway and VEGF-C/D-VEGFR-3 cascade effect [51]. In addition, tumor-derived exosome LncRNA BCYRN1 promoted tube formation and migration of HLECs, and promoted lymphangiogenesis and LN metastasis of bladder cancer. Mechanistically, LncRNA BCYRN1 activates the Wnt/β-catenin signaling pathway by upregulating WNT5A expression and synergistically enhances VEGF-C/VEGFR3 signaling axis [52].

In summary, we can see that various classical signaling pathways can participate in tumor lymphangiogenesis in a direct or indirect manner, and there is basically no specific signaling pathway. So this may be an exciting and contradictory problem. If there is no or difficult to find specific, identified and valuable key pathways or molecules in lymphangiogenesis, blocking tumor progression induced by lymphangiogenesis may face great challenges. Fortunately, since lymphangiogenesis involves various signaling pathways, the application of chemotherapy, targeted and immunotherapy drugs may inhibit tumor progression by changing the state of lymphangiogenesis to a certain extent. However, considering the modern precision medical model, more researchers still hope to seek meaningful findings.

### Molecules related to lymphangiogenesis

1. Platelet-derived growth factor-BB (PDGF-BB): as a member of the PDGF family, PDGF-BB plays a direct role in promoting lymphangiogenesis and LN metastasis, and it can activate MAP kinase activity of LECs and promote cell movement in vitro, and effectively induce the growth of lymphatic vessels in vivo [53, 54]. Inhibition of PDGF-BB can significantly reduce the ability of LEC proliferation, migration and tube formation [55]. In addition, the concentrations of VEGF-C, PDGF-BB and bFGF in hypoxic preconditioning serum (HPS) and platelet-rich plasma (PRP) were higher than those in normal serum (NS), and could significantly promote the proliferation and migration of LECs and improve the ability of lymphangiogenesis [56].
2. Angiopoietin-2 (Ang-2): Angiopoietin-2 (Ang-2) is the ligand of receptor tyrosine kinase Tie2, involved in lymphangiogenesis [57]. In the inflammatory mouse model, Ang-2 specific inhibitor L1-10 can block Ang-2 and significantly inhibit lymphangiogenesis [58]. In addition, high levels of Ang-2 are associated with tumor lymphangiogenesis and poor prognosis in non-small cell lung cancer (NSCLC) [59]. These results suggest that Ang-2, as a key regulator of lymphangiogenesis, sensitizes the lymphatic system to pathological stimuli and induces pathological lymphangiogenesis.
3. Inflammatory chemokines: Chemokines are small cytokines or signal proteins secreted by cells. Considering their ability to induce directional chemotaxis of nearby reactive cells, they can be recruited into inflammatory sites and secondary lymphoid organs through leukocyte recruitment and participate in the occurrence and progression of tumors [60]. Studies have shown that LECs not only promote lymphangiogenesis, but also have tumor chemotaxis. For example, LECs promote the invasion of lymphatic vessels by inducing the migration of cancer cells expressing CCR7 to pre-metastatic niches, and the expression of CCR7 is associated with lymphatic vascular invasion and lower survival rate [61–63]. In addition, high CCR7 expression contributes to TGF-β1-induced EMT, and promotes tumor lymphangiogenesis and LN metastasis, and is associated with poor clinicopathological and prognostic factors [64]. Another study has shown that CXCL1 secreted by lymphatic endothelial cells is involved in lymphangiogenesis and metastasis of GC by stimulating LEC migration and tube formation [65].

### Matrix microenvironment related to lymphangiogenesis

**Fig. 3:**
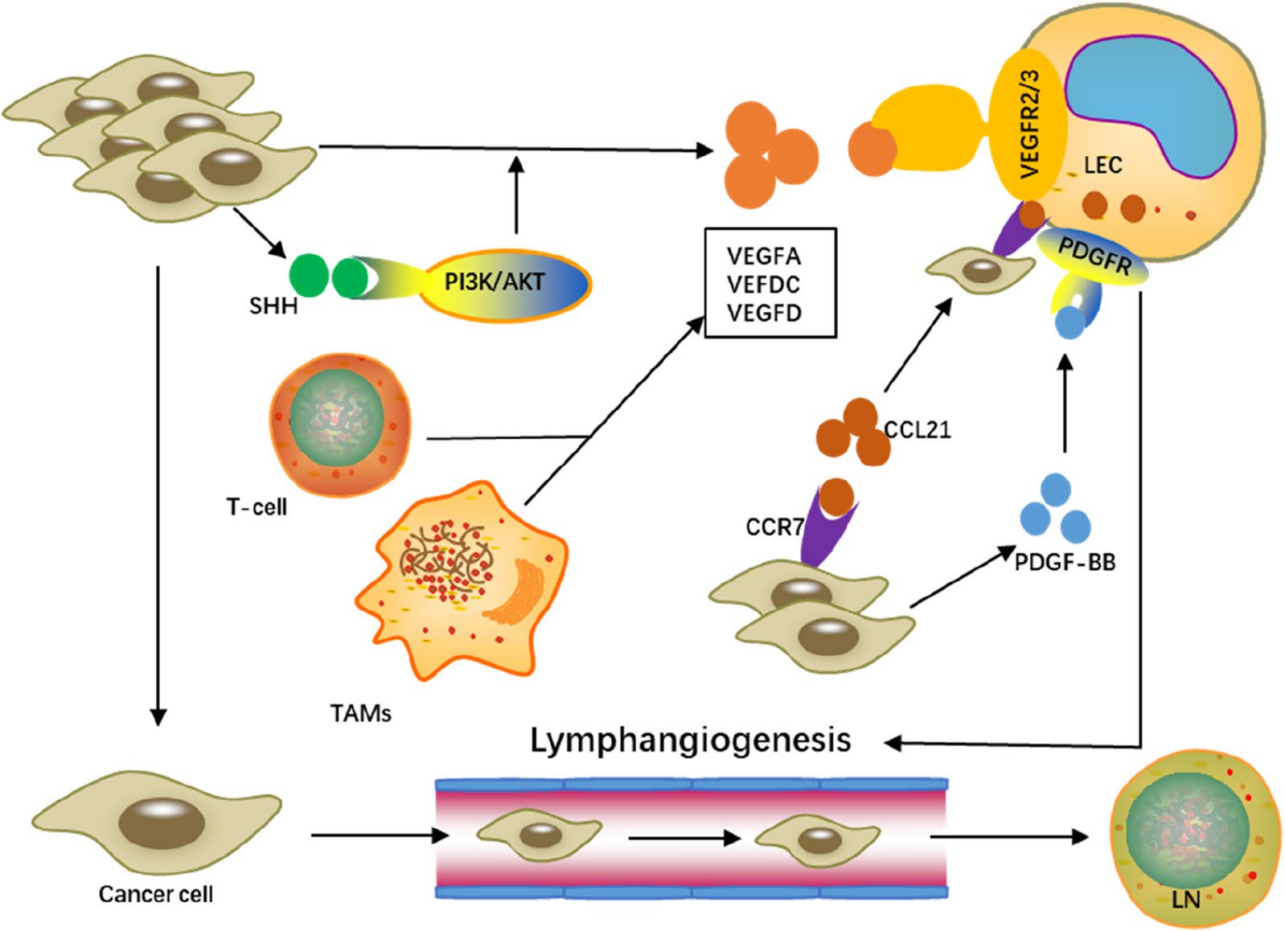
Molecular pathways that promote lymphangiogenesis in GC. SHH protein-mediated PI3K/AKT signaling pathway and TAMs and T cells in stromal microenvironment promote tumor lymphangiogenesis by inducing VEGFA/C/D expression. CCL21 expressed by LECs can induce CCR7-dependent cancer cells into lymphatic vessels. PDGF-BB secreted by tumor cells can directly induce tumor lymphangiogenesis. *SHH* sonic hedgehog, *CCL21* CC-chemokine ligand 21, *CCR7* C–C receptor 7, *PDGF-BB* platelet-derived growth factor BB, *TAMs* tumor-associated Macrophage

The matrix microenvironment is of great importance in maintaining normal tissue homeostasis or promoting tumor development. A large number of immune cells (neutrophils, lymphocytes, macrophages, mast cells, etc.) constitutes a crucial part of tumor microenvironment. Previous studies have shown that macrophages are important cells for tumor angiogenesis, supported by more evidence that they are also key participants in lymphangiogenesis [66]. PDPN is highly expressed in macrophages. PDPN combined with galectin 8 (GAL8) can activate integrin-β1 to promote LEC adhesion and lymphangiogenesis [3]. Macrophages are also an important source of VEGF-C/VEGF-D/VEGFR3. In the inflammation-induced animal models, LECs produce chemokines through LPS-Toll-like receptor 4 (TLR4)/NFKB signaling, recruit macrophages to reshape lymphatic, and enhance the expression of VEGF-C and VEGF-D, thereby promoting lymphangiogenesis [67]. Other immune cells also include mast cells promote cancer by releasing angiogenesis (VEGF-A) and lymphangiogenesis factors (VEGF-C and VEGF-D). VEGF-C/D directly mediated VEGFR3 is essential for the growth, proliferation and migration of HLEC. VEGF-A can indirectly promote lymphangiogenesis by recruiting immune cells (such as macrophages, mast cells) that produce VEGF-C and VEGF-D [68]. In addition, cancer-associated fibroblasts in the tumor microenvironment are also the main source of VEGF [69]. In cholangiocarcinoma, tumor-secreted PDGF-D can recruit and activate hepatic myofibroblasts to produce VEGF-C and VEGF-A, leading to lymphangiectasis and tumor cell infiltration, thereby inducing tumor lymphangiogenesis [70]. Moreover, hypoxia can also induce lymphangiogenesis in the tumor microenvironment, which is thought to be mostly mediated by hypoxia-inducible factor 1⍺ (HIF-1⍺) by regulating various cells in cancer-associated fibroblasts [71]. HIF-1⍺ can induce the proliferation and migration of LEC, and regulate the expression of lymph node metastasis-related growth factors and carcinogenic factors [72]. For example, adipose-derived stem cells can strongly stimulate the expression of VEGFC, VEGFR3 and PROX1 genes in the in vitro hypoxic dermal regeneration model, thereby promoting angiogenesis and lymphangiogenesis, which depends on the up-regulation of HIF-1α [73]. In addition, HIF-1α are also associated with the expression of VEGF-C, increased lymphatic vessel density and peritumoral lymphangiogenesis in breast cancer and OSCC [74, 75]. (Fig. 3 represents partial signaling pathways and molecules involved in lymphangiogenesis in GC).

### Tumor immune-related lymphangiogenesis molecules

Although tumor immunotherapy has made great progress in clinical practice, immune tolerance is still the most direct cause of immunotherapy failure in cancer patients. Recent studies have shown that LN setting can participate in immune escape by inducing immune tolerance, and increasing evidence supports that lymphatic play a key role in tumor immunosuppression [11, 76–78]. As mentioned above, the expression of VEGF-C in tumors is highly correlated with lymph node metastasis and poor prognosis of various tumors [10]. In addition, LEC can not only participate in the activation of the body’s immune system under physiological conditions, but also promote tumor progression and metastasis by expressing various peripheral tissue antigens (PTAs) to inhibit the function of immune cells [79, 80]. Mechanistically, VEGF-C can provide melanocyte-specific protein tyrosine kinase clearance and cross-presentation of antigens through LEC to induce CD8^+^ T cell dysfunction, resulting in tumor cell immune tolerance [78, 80, 81]. However, activation of CD8^+^ T cells requires antigen-presenting cells (APCs) carrying major histocompatibility class I complex (MHC-I) to present tumor-associated antigen (TAA) [82]. In addition, LEC can provide PTA to directly inhibit the maturation of DC, thereby reducing the proliferation of CD4^+^ T cells and inducing tumor tolerance [83]. IFN-γ signaling pathway in lymphatic vessels is also one of the key pathways of tumor immunosuppression. It can promote the expression of PD-L1 in LECs through JAK/STAT pathway and inhibit the accumulation of T cells, thus leading to tumor immunosuppression and immune escape [84]. For example, cervical cancer-derived exosome miR1468-5p can mediate the JAK/STAT3 pathway in LECs, promote lymphangiogenesis and disrupt T cell immunity [85]. In addition, in melanoma model, IFN-γ can promote the expression of MHC-II in LECs. MHC-II^+^ LECs can increase the number of Treg cells and reduce the number of effector T cells by presenting TAA. Moreover, the number of Treg cells was positively correlated with lymphatic vessel density [78, 86]. Additionally, MHC-II molecules in LECs can mediate CD8^+^ T cell tolerance through LAG3 [87]. Lymphatic vessels can promote tumor immune escape by reducing inflammatory cells, especially in melanoma. The density of lymphatic vessels in human melanoma is closely related to T cell infiltration and the expression of immunosuppressive molecules, indicating that tumor-associated lymphatic activation can produce tumor immunity [88]. Such as TGF-β, iNOS, IDO and NOX5, etc., can maintain peripheral tolerance to lymph node autoantigens by regulating the immune function of T cells [78, 89–91]. In addition, in colorectal cancer, the VEGFC/VEGFR3 pathway can induce the proliferation of LECs and macrophages, and VEGFR3 can also induce TAM polarization to M2 type to participate in tumor immunosuppression [92].

In summary, the current LEC-mediated tumor immune tolerance can be achieved by the following points: 1. inducing T cell dysfunction and reducing its proliferative capacity (CD8^+^ and CD4^+^ T cells); 2. LEC carrying MHC-I/II presented PTAs; 3. expression of immunosuppressive factors (TGF-β, iNOS, IDO, etc.); 4. immune checkpoints (PD-L1 and LAG-3); 5. inhibition of DC maturation.

### Tumor resistance-associated lymphangiogenesis molecules

Increased tumor resistance is a key factor in cancer progression. Previous studies have shown that Sushi Repeat Containing Protein X-linked 2 (SRPX2) acts as a tumor-promoting factor in various cancers [93–95], and the down-regulation of SRPX2 can improve the sensitivity of esophageal cancer patients to cisplatin [96]. In addition, HGF, as an important mediator of tumor lymphangiogenesis, can bind to SRPX2 to promote tumor lymphangiogenesis [97, 98]. Previous studies have also shown that SRPX2 acts as a ligand for urokinase plasminogen activator receptor (uPAR) to regulate endothelial cell migration and tube formation [99]. Subsequently, Sasahira et al. found that SRPX2, as a downstream gene of LEMD1, may induce cisplatin resistance and lymphangiogenesis in oral squamous cell carcinoma (OSCC) through uPAR and/or HGF [100]. In addition, Shimomura et al. found that Non-SMC Condensin I Complex Subunit H (NCAPH) was also involved in lymphangiogenesis and tumor resistance in OSCC [101].

### Effect of lymphangiogenesis on tumor metastasis

Previous studies have clearly reported that lymphangiogenesis plays a crucial role in promoting tumor progression and metastasis. The expression of lymphangiogenesis factor, VEGF-C and higher lymphatic vessel density are related to the progression, metastasis and low survival rate of tumor patients [102–104]. For example, fatty acid synthase (FASN) is up-regulated in cervical cancer (CC), and it is associated with LN metastasis. Mechanistically, FASN induces lymphangiogenesis by secreting PDGF-AA/IGFBP3, thereby promoting LN metastasis [105]. S1PR1 on tumor-associated macrophages promotes lymphangiogenesis and tumor metastasis in breast cancer patients through the NLRP3/IL-1 pathway. The expression of NLRP3 is related to LN invasion, metastasis and prognosis of patients [106]. CircEHBP1 is significantly up-regulated in bladder cancer (BC), and it is associated with LN metastasis and poor prognosis in patients with BC. Mechanistically, circ EHBP1 promotes VEGF-D expression by mediating TGF-β/SMAD3 signaling pathway, thereby inducing lymphangiogenesis and lymphatic metastasis of BC [107]. Exosome-mediated lymphangiogenesis is also considered to be an important driver of LN metastasis [108–111]. For example, exosome-derived long non-coding RNA (LNMAT2) can induce LECs to obtain enhanced tube formation and migration, resulting in LNM in bladder cancer [108]. Cervical cancer-derived exosome miR-221-3p promotes lymphangiogenesis and lymphatic metastasis by targeting VASH1 [109]. Exosomes derived from melanoma and colorectal cancer have also been shown to promote LN metastasis by remodeling lymph nodes and lymphatic networks [110, 111]. In addition to the above LN metastasis, lymphangiogenesis may also be associated with distant metastasis. An animal model-based study by Hirakawa showed that VEGF-C first induced the expansion of lymphatic network in sentinel LNs before tumor metastasis. When the tumor cells metastasize to the sentinel LN, the lymphangiogenesis in the corresponding site increases. Moreover, in mice with sentinel LN metastasis, tumors expressing VEGF-C were more likely to metastasize to other organs, such as distal LNs and lungs [112]. In addition, several recent studies have also shown that, as mentioned above, LN setting can induce immune tolerance, thereby promoting distant metastasis in the mouse model established by melanoma cells [11].

### Effect of lymphangiogenesis on gastric cancer metastasis

LN metastasis is an important factor affecting the prognosis of GC, and lymphangiogenesis factors secreted by cancer cells have obvious advantages in promoting lymphangiogenesis and tumor cell metastasis [69, 113]. For example, Ma C et al. [114] found that kallistatin was down-regulated in GC tissues, metastatic LNs and plasma, and its plasma level was negatively correlated with LN metastasis stage. Mechanistically, kallistatin down-regulates VEGF-C expression and secretion by mediating NF-κB signaling, thereby inhibiting tumor lymphangiogenesis and lymphatic metastasis. Plasma oxidized low density lipoprotein (oxLDL), a risk factor for tumorigenesis in patients with abnormal lipid metabolism, can also mediate NF-κB signaling to promote lymphangiogenesis and lymphatic metastasis in GC [115]. Sterol oxygen-acyltransferase 1 (SOAT1) is highly expressed and is associated with advanced tumors, LN metastasis and poor prognosis in GC. Mechanistically, SOAT1 promotes the expression of VEGF-C, induces lymphangiogenesis and LN metastasis by regulating the expression of cholesterol metabolism genes SREBP1 and SREBP2 [116]. In addition, exosomal CD44 mediates yap-cpt1a-mediated FAO reprogramming is also considered to be an important driver of lymphangiogenesis and LN metastasis [117]. Tumor-associated macrophages (TAMs) are also involved in tumor lymphangiogenesis and are closely related to serosal invasion, LN metastasis and tumor stage. The expression of VEGF and VEGF-C in macrophages is up-regulated and positively correlated with MVD and LVD [118]. At the same time, tumor-associated neutrophils (TANs) in regional LNs can also enhance lymph by enhancing lymph [119].

### Gastric cancer-related lymphangiogenesis molecules or markers

Lymphangiogenesis, the formation of new lymphatic vessels induced by tumor, is directly related to the degree of metastasis of solid tumors in lymph nodes [102–104]. Lymphatic vessel density (LVD) is a quantitative measurement of tumor lymphangiogenesis measured by direct counting of lymphatic vessels. It has been reported that high LVD in GC is associated with regional LN metastasis and poor prognosis [120, 121]. However, the significance of intratumoral lymphatic vessel density (I-LVD) and peritumoral lymphatic vessel density (P-LVD) remains controversial in GC. Pak et al. [122] evaluated the I-LVD and P-LVD samples of 66 patients with radical gastrectomy and found that I-LVD was positively correlated with diffuse GC subtype, tumor stage, lymphatic vascular invasion, LN metastasis and OS. P-LVD was associated with lymphovascular invasion, LN stage and DFS. The results showed that both LVDs contributed to the progression and prognosis of GC. However, Wang et al. [123] determined the intratumoral and peritumoral lymphatic vessel density by immunohistochemistry (IHC) and found that P-LVD was significantly correlated with LN metastasis, lymphatic invasion, VEGF-C, VEGF-D and VEGFR-3 expression in peritumoral tissues, and was an independent risk factor for LN metastasis, but there was no significant association between the above variations and I-LVD.

Although the role of lymphangiogenesis remains unclear in GC, studies have shown that lymphatic vessel invasion is significantly associated with LN metastasis, and the prognosis of patients with lymphatic vessel invasion is relatively poor in GC. Here, we summarize some molecular findings on LN metastasis and lymphangiogenesis in gastric cancer (Table 1).

**Table 1:** Gastric cancer-related lymphangiogenesis molecules

Year	Molecule	Function	Mechanism or associated molecules	References
2010	NRP2	Accelerator	NRP2/VEGF-C/VEGFR3	[20]
2011	Shh	Accelerator	Shh/PI3K/Akt/EMT,MMP-9	[29]
	Id-1	Inhibitor	–	[140]
	HGF	Accelerator	HGF/c-Met	[25]
	iNOS	Accelerator	LVD	[137, 138]
2012	EphA3	Accelerator	–	[129]
	CXCL1	Accelerator	CXCL1/NF-ҡB, FAK-ERK1/2-RhoA, F-actin	[65]
	SOX 18	Accelerator		[141]
2013	CNTN-1	Accelerator	CNTN-1/VEGF-C, VEGFR-3	[142]
	ROSI	Inhibitor	ROSI/VEGF-C, VEGFR-3	[143]
2014	ECM1	Accelerator	ECM1/LMVD	[144]
	TP	Accelerator	–	[145]
	KAI1	Inhibitor	KAI1/MVD, LVD	[146]
2015	MACC1	Accelerator	MACC1/HGF/c-Met/VEGF-C/D	[27]
	claudin 4	Inhibitor	–	[147]
	IL-8	Accelerator	IL-8/VEGF-C, VEGF-D, VEGFR-3	[148]
2016	IL-6	Accelerator	IL-6/JAK-STAT3-VEGF-C	[44]
	RNF180	Inhibitor	RNF180/VEGF-C, D, CXCL7	[136]
	TAMs	Accelerator	VEGF-C, LVD	[118]
2017	PROX1	Accelerator	PROX1/b-catenin, ERK1/2, p38, JNK	[134]
	KLHL6	Accelerator	KLHL6/HGF, MMP-2和VEGF-C	[135]
2018	Kallistatin	Inhibitor	Kallistatin/NF-ҡB/VEGF-C	[114]
	COX-2	Accelerator	COX-2/VEGF-C	[127]
	PTBP3	Accelerator	PTBP3/CAV1	[131]
2019	oxLDL	Accelerator	oxLDL/NF-ҡB/VEGF-C	[115]
	Macrophage	Accelerator	VEGF-A/VEGF-C/VEGF-D	[68]
	HMGB1	Accelerator	HMGB1/VEGF-D	[149]
	HOXB9	Accelerator	HOXB9/VEGF-D	[150]
	MicroRNA-7	Inhibitor	MicroRNA-7/NF-κB/VEGF	[151]
2020	GREM1	Accelerator	GREM1/VEGFC, PDPN, LYVE	[152]
	BMPRs	Accelerator	–	[42]
	LncRNA-HNF1A-AS1	Accelerator	LncRNA-HNF1A-AS1/miR-30b-3p/PI3K/AKT	[153]
2021	SOAT1	Accelerator	SOAT1/SREBP1, SREBP2/VEGF-C	[116]
2022	hsa_circ_0000437	Accelerator	hsa_circ_0000437/HSPA2-ERK	[131]
	lncRNA ANRIL	Accelerator	lncRNA ANRIL/VEGF-C/VEGFR-3	[154]

Lymphangiogenesis has a positive effect on LN metastasis of GC. VEGF-C and VEGF-D are key regulators of lymphangiogenesis [10, 122]. The binding site of SP1 is considered to be a specific promoter of VEGF-C [124]. MACC1 can directly or indirectly bind to the SP1 site [125], which will strongly indicate that MACC1 plays a catalytic role in regulating lymphangiogenesis. Sun et al. found that MACC1 promotes lymphangiogenesis and LN metastasis of GC by upregulating VEGF-C/D expression [27]. Previous studies have shown that cyclooxygenase-2 (COX-2) promotes lymphangiogenesis by upregulating VEGF-C [126]. Subsequently, A mouse model study has shown that COX2 inhibitors can induce tumor cell apoptosis and anti-proliferative effects by reducing the expression of VEGF-C and inhibiting tumor lymphangiogenesis, thus exhibiting significant anti-tumor activity [127]. The Eph/ephrin system also have an important role in lymphangiogenesis. For example, the Eph / ephrin system is involved in the internalization of VEGFR3 and VEGFR2 and controls lymphangiogenesis and reconstruction of lymphatic vessels during tumorigenesis and inflammation [128]. EphA3 expression is associated with VEGF and patient prognosis in GC [129]. Previous studies have shown that AKT and ERK pathways are involved in lymphangiogenesis and LN metastasis [130]. Shen et al. [131] found that hsa_circ_0000437 promoted the invasion, migration and tube formation of HLEC in vitro, and promoted lymphangiogenesis and LN metastasis in popliteal LN metastasis model in vivo. Mechanistically, hsa_circ_0000437 induces LN metastasis through the HSPA2-ERK signaling pathway independent of VEGF-C. Polypyrimidine Tract Binding Protein 3 (PTBP3) is an essential RNA-binding protein that functions in RNA splicing, 3ʹ-end processing, and translation [132]. Chen et al. [131] found that PTBP3 was significantly up-regulated in LN metastasis of GC, and patients with high PTBP3 expression have a shorter survival time. In addition, in a mouse xenograft tumor model, knockout of PTBP3 inhibits tumor lymphangiogenesis and metastasis to regional LNs. Prospero Homeobox 1 (PROX1) is a tumor suppressor gene or oncogene in tumor types [133]. Park et al. [134] found that knockdown of PROX1 inhibited tumor cell proliferation, reduced LECs invasion and tube formation, and increase the expression of VEGF-C, VEGF-D, COX -2 in GC cells. Mechanistically, PROX1 can induce dephosphorylation of β-catenin and phosphorylation of ERK1/2, p38 and JNK to participate in tumor cell proliferation and lymphangiogenesis. KLHL6 protein was much higher than that in atrophic gastritis, intestinal metaplasia and dysplasia in benign gastric disease specimens in GC tissues, and KLHL6 significantly enhanced the expression of proliferation-related genes HGF, MMP-2 and VEGF-C in GC cells [135]. Ring finger protein (RNF) 180 was down-regulated in GC tissues and cells, and was negatively correlated with the number of metastatic LN. Deng’s experiments in cells and animals showed that RNF180 not only inhibited cell proliferation, migration and invasion, but also inhibited tumor growth and lymphangiogenesis. In addition, RNF180 also down-regulated the expression of HGF, VEGF-C/D and CXCL7 [136]. Research shown that increased expression of inducible nitric oxide synthase plays a key role in tumor progression. It mainly exists in the cytoplasm and is highly expressed in GC tissues and is associated with LN metastasis, vascular invasion, distant metastasis, TNM stage and poor survival rate. In addition, inducible nitric oxide synthase positive patients showed higher microvascular density and lymphatic vessel density [137, 138].

MicroRNA (miRNA) is a class of regulatory non-coding RNA, which is related to the progression of GC. Given that VEGF-C is a key regulator of lymphangiogenesis, Yang et al. [139] further validated microarray-based identification of differentially expressed miRNAs and RT-PCR in VEGF-C-transfected and non-transfected gastric cancer cells. The results showed that in VEGF-C transduced GC cells, 47 were up-regulated and 42 were down-regulated. In addition, in patients with positive LN metastasis of primary GC, the up-regulated miRNAs included miR-648, miR-5002-3p, miR-4754, miR-4460-5p, miR-4491, miR4252, miR-5007-3p and miR-647; the down-regulated miRNAs included miR-3178, miR-593-5p, miR-4485, miR-135a-3p, miR-17, miR-1469 and miR-124-5p. (Other molecular markers Reference Table 1.)

### Drugs targeting angiogenesis in gastric cancer

GC is the most common malignant tumor of the digestive system, and the prognosis of traditional surgical treatment and chemotherapy is poor. However, molecular targeted therapy is a research hotspot in the field of tumor therapy in recent years. Among them, the application of anti-angiogenic drugs in the comprehensive treatment of gastric cancer has made great progress, including monoclonal antibodies targeting VEGF, tyrosine kinase receptor inhibitors, and antibodies targeting VEGFR. In addition, FGF (fibroblast growth factor) and FGF receptor, PDGF and PDGF receptor, ANG and TIE2 receptor pathways are also involved in angiogenesis of malignant tumors and can also be used as targets for anti-angiogenesis drugs. The following two drugs are currently approved by FDA for targeted anti-vascular therapy of GC.

Ramucirumab: Ramucirumab is an antagonist of VEGFR2. It can specifically bind to VEGFR2 and block the coordination of VEGF ligands, VEGF-A, VEGF-C and VEGF-D. Therefore, Ramucirumab inhibits the activation of VEGFR 2 stimulated by ligands, thereby inhibiting ligand-induced proliferation and migration of human endothelial cells. Based on the excellent performance of its anti-angiogenic drugs, it has been approved by the FDA for second-line treatment of gastric cancer [155]. Moreover, RAINBOW-Asia studies have shown that the efficacy and safety of Ramucirumab in Asian populations, especially in Chinese populations, have been further confirmed [156].

Apatinib: An oral small molecule tyrosine kinase inhibitor that selectively inhibits VEGFR2-induced endothelial cell migration and proliferation, thereby preventing the formation of new blood vessels. Apatinib is the world's first small molecule anti-angiogenic targeted drug that has been shown to be safe and effective in AGC, and a large number of clinical studies have shown that Apatinib can significantly prolong the survival of patients with advanced gastric cancer by inhibiting the formation of new blood vessels in tumor tissue [157].

### Targeted anti-angiogenesis drugs for other tumors

Sorafenib: Sorafenib is the first multi-target kinase inhibitor approved for the treatment of liver cancer, kidney cancer, thyroid cancer. Sorafenib can simultaneously inhibit a variety of intracellular and cell surface kinases, including RAF kinase, VEGF-2, VEGF-3, PDGFR-β, KIT and FLT-3. Not only can it directly inhibit tumor growth through KIT and FLT-3 inhibition of RAF/MEK/ERK signaling pathway, but also indirectly blocking tumor angiogenesis by blocking VEGFR and PDGFR with a dual anti-tumor effect [158].

Lenvatinib: Lenvatinib is a TKI for VEGFR1-3, PDGFR and FGFR. For first-line treatment of patients with advanced liver cancer [159]. Besides, lenvatinib also significantly reduced LVD in metastatic nodules after resection of primary lung cancer [160]. Moreover, it can also inhibit VEGF and FGF-driven proliferation and angiogenesis mechanisms [161].

Bevacizumab: Bevacizumab is an anti-VEGF monoclonal antibody that specifically binds to VEGF-A and blocks the angiogenic cell pathway. It is the world’s first approved anti-tumor angiogenesis targeted drug and the first recombinant humanized anti-VEGF monoclonal antibody. Among them, bevacizumab has shown good results in the anti-tumor treatment of gastric cancer [162].

## Conclusions and prospects

Although LN metastasis and lymphangiogenesis in malignant tumors have been extensively studied, the depth of research in gastric cancer is far from adequate. In view of the poor prognosis of patients with LN metastasis of GC, the following points may need to be specifically studied: (1) to find efficient LEC markers for gastric cancer; (2) to determine the specific role of LECs in the progression of gastric cancer; (3) to find lymphatic molecular targets to improve treatment outcomes.

Identification of high-efficiency LEC markers for GC: a variety of proteins have been identified on LEC, including PROX1, SOX18, NRP2, and VEGFR3. Although the above protein markers are associated with lymphangiogenesis in GC, only two proteins, LYVE1 and podoprotein, have been routinely monitored in cancer in the past 10 years to identify lymphatic vessels and have been used for immunohistochemistry or immunofluorescence. Therefore, it is feasible to develop efficient biomarkers or their combinations to improve the diagnosis and precise treatment of diseases.

The specific role of LECs in the progression of GC: as previously mentioned, LECs can participate in various adverse prognosis of cancer through a variety of molecules (VEGFC, VEGFR3 and chemokines, etc.) or signaling pathways (TGF-β, etc.). However, lymphatic vessels may play a contradictory role in tumor progression, not only allowing metastasis, but also enhancing key checkpoints in immune recognition and anti-tumor responses. For example, a previous study based on a mouse melanoma model showed that blocking VEGFR3 could reduce the tumor infiltration of naive T cells and inhibit the therapeutic effect of tumor. In addition, in human metastatic melanoma, VEGF-C-mediated lymphangiogenesis enhances immunotherapy. Thus, the crosstalk between LEC, tumor cells, and anti-tumor immunity may determine tumor progression [163]. Therefore, it is necessary to determine the specific role of LECs in the progression of gastric cancer for the next development of precise targeted therapy.

Looking for lymphatic molecular targets to improve treatment outcomes: to date, increasing evidence has shown that lymphatic endothelial cells maintain important functions in the progression of a variety of malignant tumors and are highly clinically significant. For example, LECs can induce chemotherapy resistance, immune tolerance and local or distant metastasis of tumor cells. Therefore, by exploring the specific role of LECs in tumors, we can develop targeted research programs to identify new molecular targets to improve the response of the LEC pathway to precise treatment of cancer.

In summary, in order to develop a treatment for tumor cell progression induced by targeted LECs, it is necessary to identify high-efficiency markers related to lymphangiogenesis and address the necessary hazards of lymphangiogenesis in GC. So it is necessary to further study the lymphatic involvement area in GC.

## Data Availability

All data and materials in our study are available upon reasonable request.
